# Supplementary material for: IL-22-dependent dysbiosis and mononuclear phagocyte depletion contribute to steroid-resistant gut graft-versus-host disease in mice
Source: Nat Commun. 2021 Feb 5;12:805. doi: 10.1038/s41467-021-21133-3 (PMC7865028; doi:10.1038/s41467-021-21133-3)
Supplement: Supplementary file 1 — Supplementary Information [file 41467_2021_21133_MOESM1_ESM.pdf]

Supplementary Information

IL-22-dependent dysbiosis and mononuclear phagocyte depletion contribute to steroid-resistant gut graft-versus-host disease in mice

Qingxiao Song, Yuan-Zhong Chen, Defu Zeng, *et al.*

a

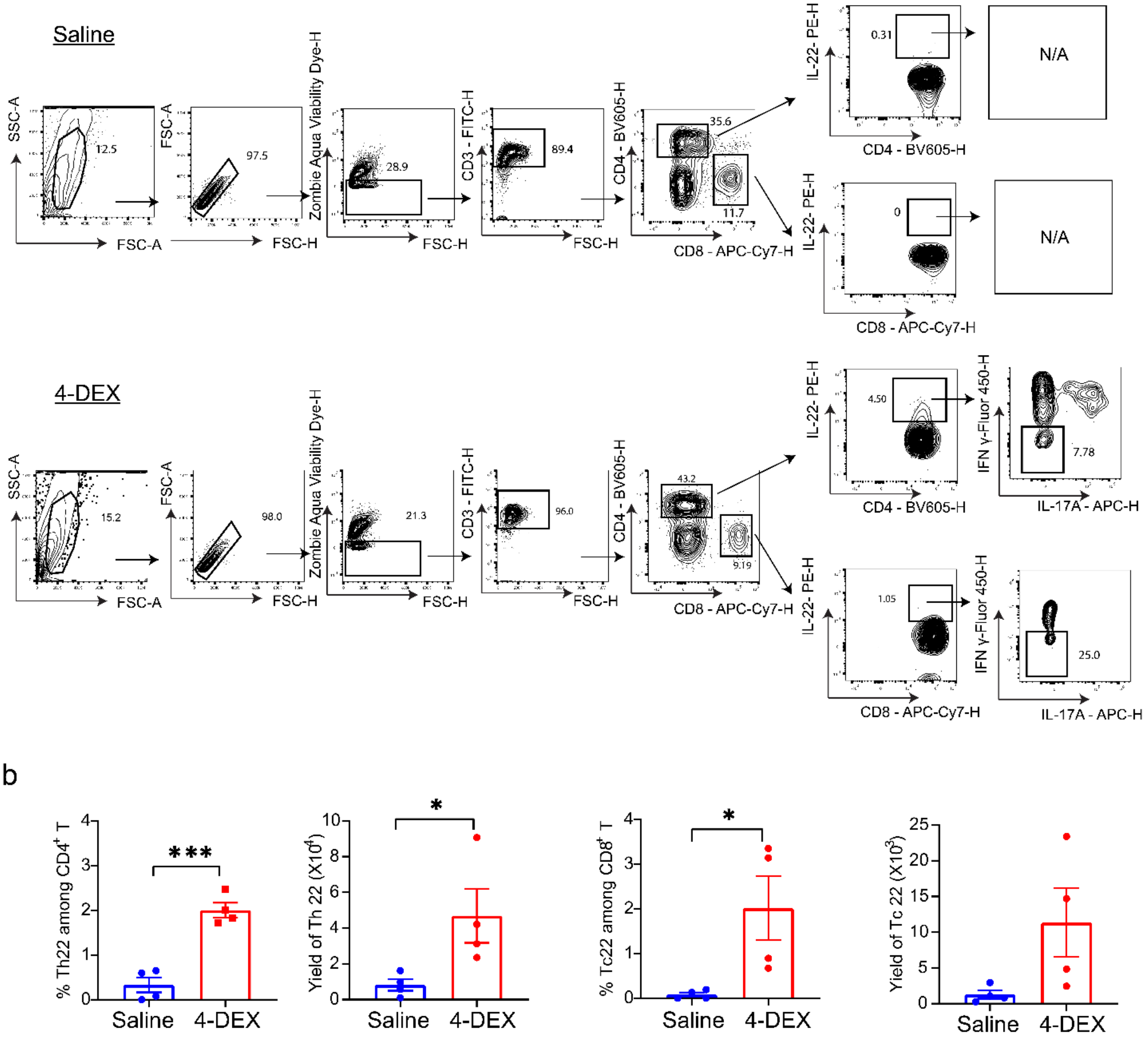

Supplementary Fig. 1. Prolonged dexamethasone treatment augments the expansion of human Th/Tc22 cells in the gut tissues of Xeno-GVHD recipients. RAG2$^{-/-}$/IL-2R$\gamma^{-/-}$ mice were given a single 350 cGy fraction of total body irradiation before injection of 30×10^6 human PBMC on the same day. On days 3, 6, 9, 12 after HCT, recipients were given a total of 4 injections of dexamethasone (DEX) at 5mg/kg or saline. On day 15 after HCT, gut tissue of recipients was analyzed for Th/Tc22 cells. **(a)** Flow cytometry gating strategies for human Th/Tc22 cells. **(b)** Means ± SEM of percentages and yields of Th/Tc22 are shown. n=4 combined from two replicated experiments. Each dot represents one mouse. Unpaired two-tailed Student's t test was used to compare means. **b**, ***p = 0.0004(%Th22), *p = 0.0461(Yield of Th22). *p = 0.0356 (%Tc22).

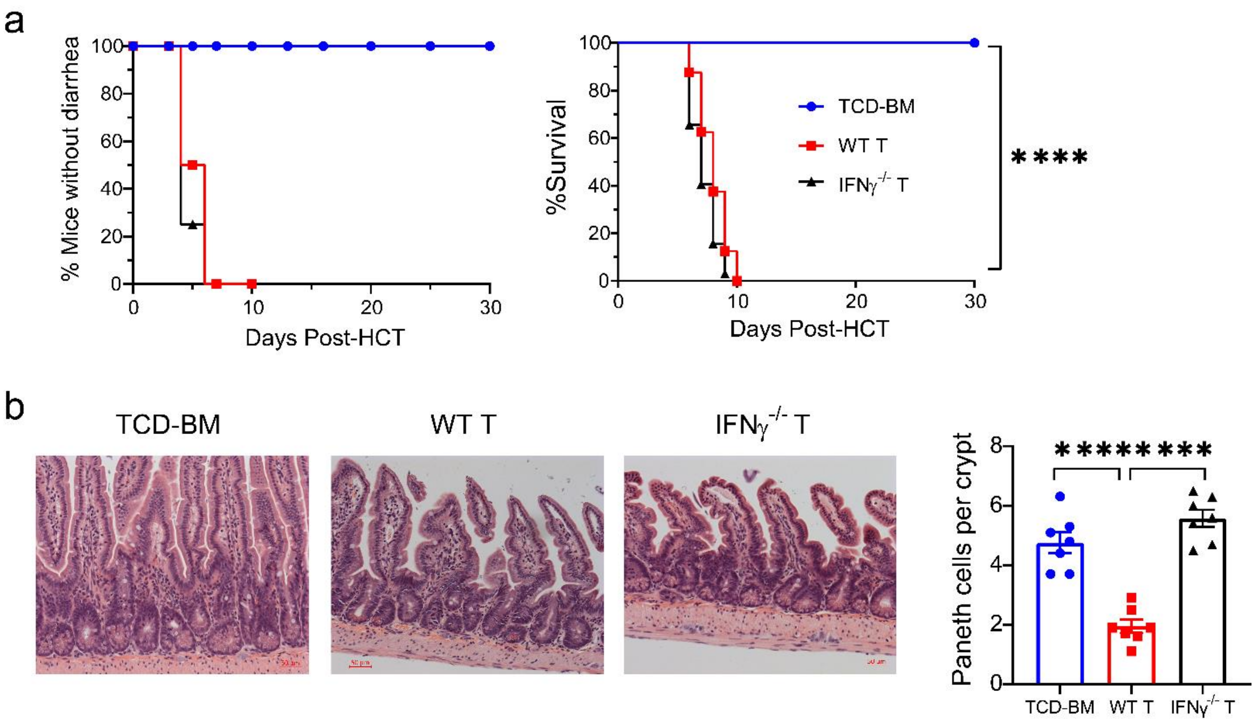

Supplementary Fig. 2. Wild-type but not IFN-γ$^{-/-}$ CD4^{+} and CD8^{+} T cells induce Gut-aGVHD with damage to Paneth cells. Lethally irradiated BALB/c recipients were engrafted with TCD-BM (2.5x10^{6}) from WT C57BL/6 donors with or without additional whole splenocytes containing 1.5x10^{6} CD4^{+} and CD8^{+} T cells from WT or IFN-γ $^{-/-}$ C57BL/6 donors. Recipients were monitored for clinical signs of aGVHD for up to 30 days. **(a)** Recessive curves of %Mice without diarrhea and %Survival are shown. n=8. **(b)** On day 7 after HCT, small intestine tissue was harvested for H&E staining. One representative microphotograph (Original magnification 200x) and Means ± SE of the numbers of Paneth cell per crypt are shown, n=7. All results are combined from 2 replicates. Each dot represents one mouse, one-way ANOVA was performed compare the means of Paneth cell per crypt between different groups. Log-rank test was performed with two-tailed *p*-value to compare survival. a, **** $p < 0.0001$; b, **** $p < 0.0001$.

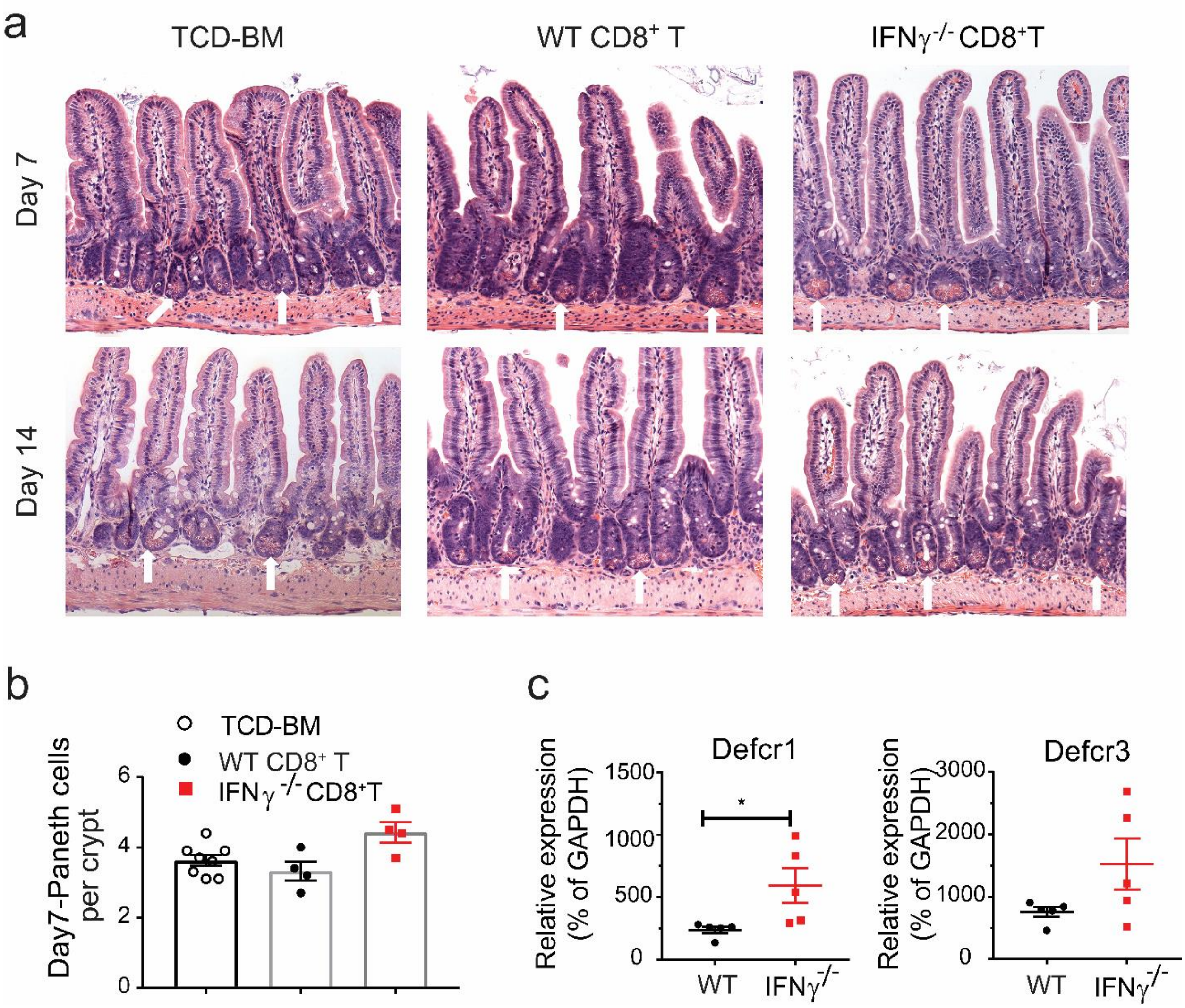

Supplementary Fig. 3. IFN-γ$^{-/-}$ donor CD8^{+} T cells induce Gut-aGVHD without damaging Paneth cells. Lethally irradiated BALB/c recipients were engrafted with TCD-BM with or without splenocytes from WT or IFN-γ$^{-/-}$ C57BL/6 donors, as described in Fig.4a. (**a)** Representative microphotographic photos (scale bar 50μm, original magnification 200x) of ileum H&E staining at 7 and 14 days after HCT, with arrows pointing to Paneth cells, n=4. **(b)** At day 7 after HCT, Paneth cells in the crypts were counted with enlarged photos on the computer screen. Means ± SE of Paneth cell numbers/Crypt of recipients given TCD-BM alone, WT CD8^{+} T, or IFN-γ$^{-/-}$CD8^{+} T are shown, n=4 (WT CD8^{+} T & IFN-γ$^{-/-}$ CD8^{+} T), n=8 (TCD-BM), combined from two replicate experiments. **(c)** At day 7 after HCT, ileal tissue from recipients given WT CD8^{+} T cells or IFN-γ$^{-/-}$ CD8^{+} T cells were tested for mRNA expression of Defensins (Defcr1 and Defcr3). Means ± SE of 5 recipients/group are shown. Each dot represents one mouse. Unpaired two-tailed Student's t tests were used to compare means. **c**, *p<0.0377.

IgG

Anti-IL-22

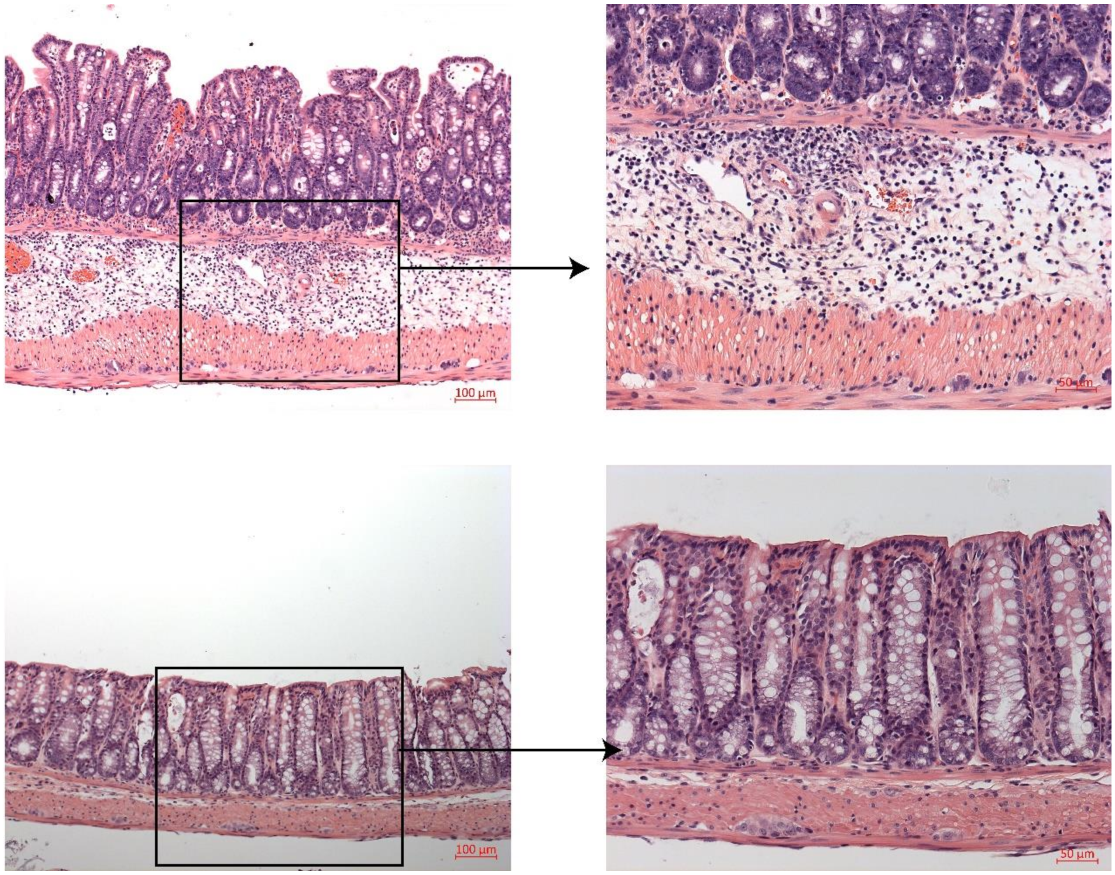

Supplementary Fig. 4. Neutralization of IL-22 prevents Gut-aGVHD induced by IFN-γ$^{-/-}$ donor CD8^{+} T cells. Lethally irradiated WT BALB/c recipients were engrafted with splenocytes containing T cells (1.5x10^{6}) from IFN-γ$^{-/-}$ C57BL/6 donors together with TCD-BM (2.5x10^{6}) from WT C57BL/6 donors and treated with anti-CD4 mAb (500µg/mouse) on the day of HCT**.** Recipients were also treated with anti-IL-22 mAb or control mouse IgG (150µg/mouse), every other day from days 0 to 6 after HCT**.** At day 14 after HCT, colon sections were stained with H&E. The boxed areas are shown for colonic epithelial cell damage and lamina propria infiltration in recipients treated with control mouse IgG but not in recipients treated with anti-IL-22. Micrograph photos are representative from 1 of 4 replicate experiments.

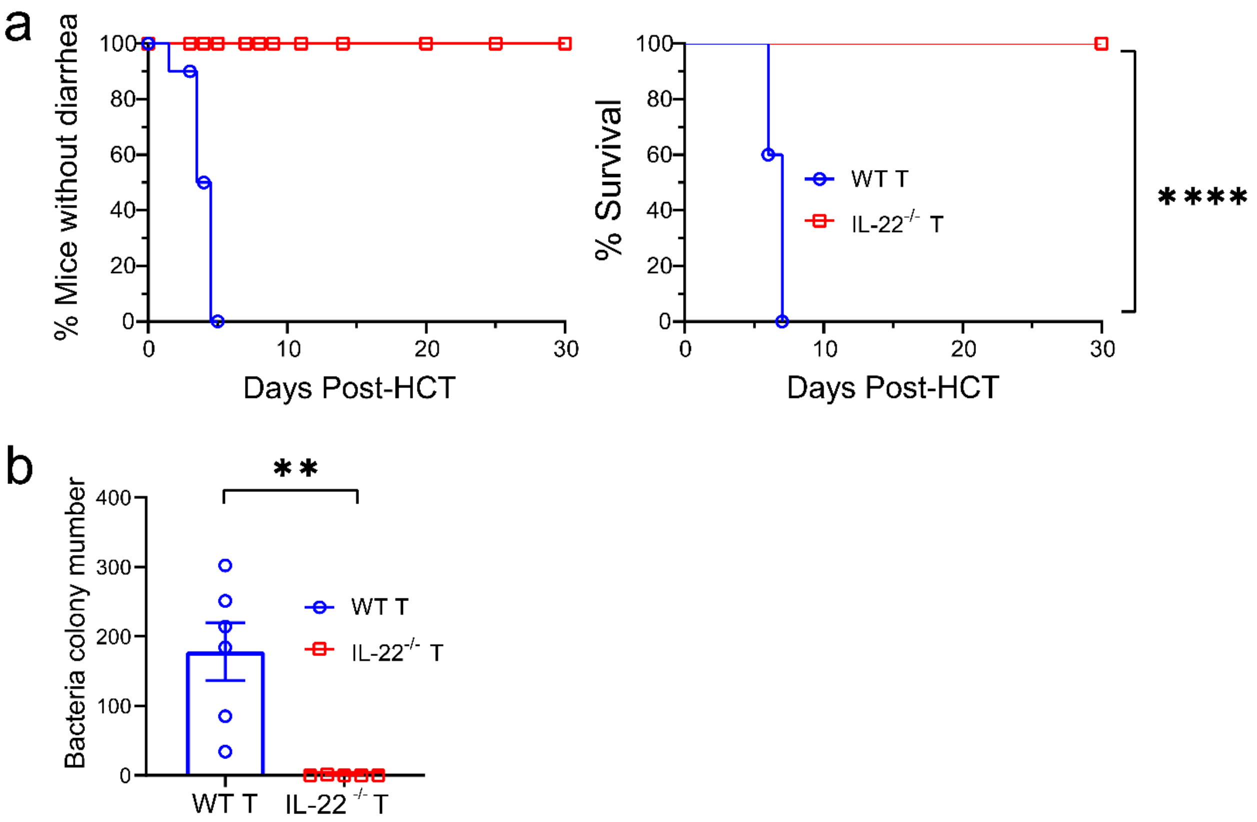

Supplementary Fig. 5. IL-22 from alloreactive T cells induces lethal aGVHD. Lethally irradiated BALB/c recipients were engrafted with splenocytes containing T cell ($1.5x10^6$) from WT or IL-22$^{-/-}$ C57BL/6 donors together with TCD-BM cells ($2.5x10^6$) from WT C57BL/6 donors. Recipients were treated with anti-CD4 Ab (1mg/mouse) on day 0 and daily anti-IFN-γ (1mg /mouse) from days 0 to 5 after HCT. Recipients were monitored for clinical signs of aGVHD for up to 30 days. **(a)** Recessive curves of %Mice without diarrhea and %Survival among total mice are shown; n=10. **(b)** At days 6 after HCT, liver suspensions were cultured for bacterial colony formation. Means ± SE of bacterial colonies/culture are shown, n=5 (IL-22$^{-/-}$ T), n=6 (WT T). Each dot represents one mouse. Unpaired two-tailed Student t tests were used to compare means. Log-Rank test with two-tailed *p*-value was used to compare survival. **a**, **** $p < 0.0001$; **b**, ** p =0.0037.

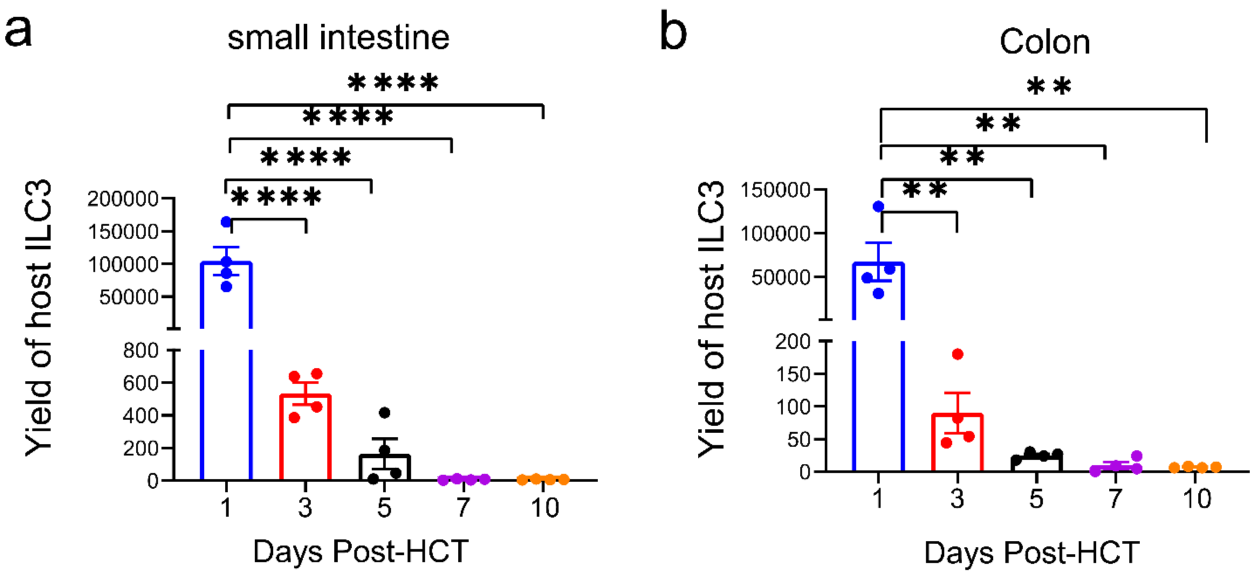

Supplementary Fig. 6. IL-22-producing host-type ILC3 cells are eliminated before Gut-aGVHD onset. Lethally irradiated BALB/c recipients were given splenocytes from IFN-$\gamma^{-/-}$ C57BL/6 donors together with TCD-BM from WT C57BL/6 donors as described in Fig.4a. Yields of ILC3 cells in the small intestine **(a)** and colon **(b)** on day 1, 3, 5, 7 and 10 days after HCT are shown. Mean ± SE, n=4. All results combined from two replicate experiments. Each dot represents one mouse. One-way ANOVA was performed to compare the means between different groups. **a**, **** $p < 0.0001$; **b**, ** $p = 0.0016$.

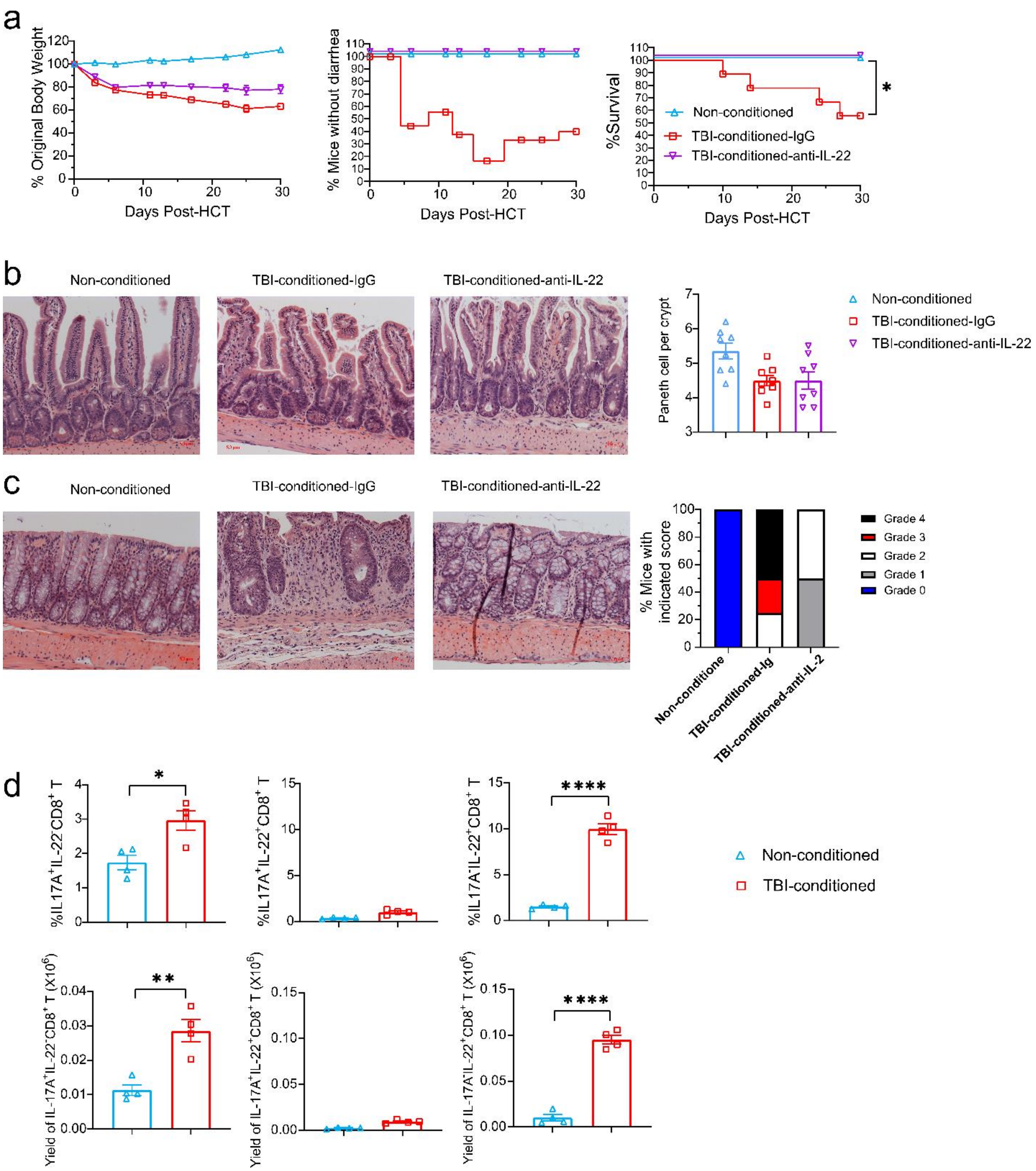

Supplementary Fig. 7. TBI-conditioning is required for induction of Gut-aGVHD by Tc22 cells in a haploidentical HCT model. CB6F1 recipients with or without TBI-conditioning (1300cGy) were engrafted with CD4$^+$ T-depleted splenocytes containing 2x10^6 CD8$^+$ T cells from IFN-γ$^{-/-}$ C57BL/6 donors together with 2.5x10^6 TCD-BM from WT C57BL/6 donors. The recipients given TBI-conditioning were treated with IL-22 mAb or control IgG (150μg/mouse) on days 0, 2, 4 and 6 after HCT. Recipients were monitored for clinical signs of aGVHD for up to 30 days. **(a)** Plots of Means ± SEM of %Original bodyweight change, recessive curves of %Mice

without diarrhea, and %Survival are shown, n=8 (Non-conditioned & TBI-conditioned-anti-IL-22), n=9 (TBI-conditioned-IgG). **(b & C)** On day 30 after HCT, small intestine and colon sections stained with H&E were analyzed by histopathology. One representative of micrograph (original magnification, x200) of small intestine **(b)** and colon **(c)** and means ± SEM of GVHD pathological scores are shown; n=8 biologically independent mice. **(d)** Mesenteric lymph node (MLN) from TBI-conditioned or non-conditioned recipients were harvested on day 30 after HCT. Means ± SE of percentages and yield of $IL17A^{+}IL22^{-}$, $IL17A^{+}IL22^{+}$ and $IL17A^{-}IL22^{+}$ $CD8^{+}$ T cell subsets are shown. n=4. All results are combined from two replicates. Each dot represents one mouse. Log-rank test with two-tailed *p*-value was used for comparison of survival. Unpaired two-tailed Student t tests were used to compare means between groups. a, * $p < 0.0133$; d, *p=0.0129, **p=0.0028, ****p<0.0001.

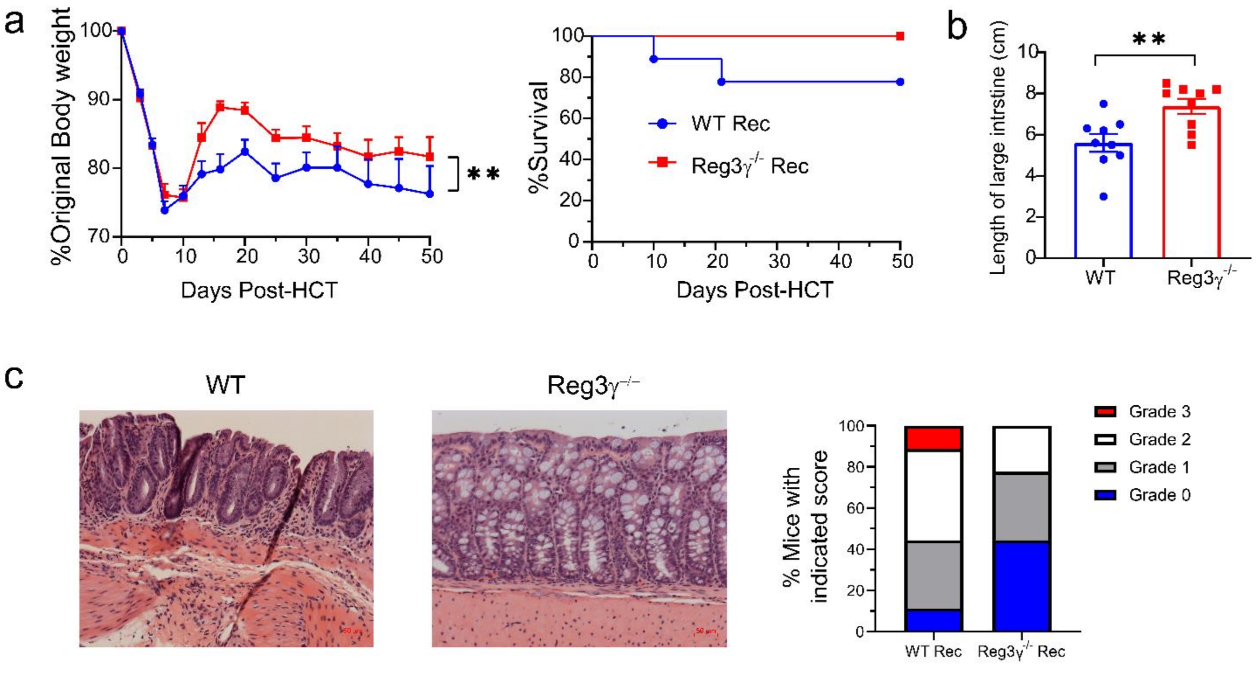

Supplementary Fig. 8. Reg3γ deficiency in recipients ameliorates SR-Gut-aGVHD. Lethally irradiated WT or Reg3γ$^{-/-}$ C57BL/6 recipients were given splenocytes contained 1.5x10^{6} T cells and 2.5x10^{6} TCD-BM cells from WT BALB/c donors. Recipients were treated with DEX (5mg/kg) on day 3, 10, 15 and 20 after HCT and were monitored for clinical signs of GVHD for up to 50 days. **(a)** Plots of Mean ± SEM of %Original bodyweight at each time point and recessive curves of %Survival among total mice are shown. n=9. **(b)** On day 50 after HCT, the length of colon was measured, and means ± SE are shown. n=9. **(c)** At day 50 after HCT, H&E stained colon sections were analyzed by histopathology. One representative microphotograph (Original magnification 200x) and the distributions of histopathology scores are shown. n=9 biological independent mice. All results are combined from 2 replicates experiments. Each dot represents one mouse. Unpaired two-tailed Student t tests were used to compare means. Nonlinear regression with two-tailed *p*-value was used to compare body weights between 2 groups. **a,** ** *p*= 0.0045; **b**, ** *p*= 0.0012.

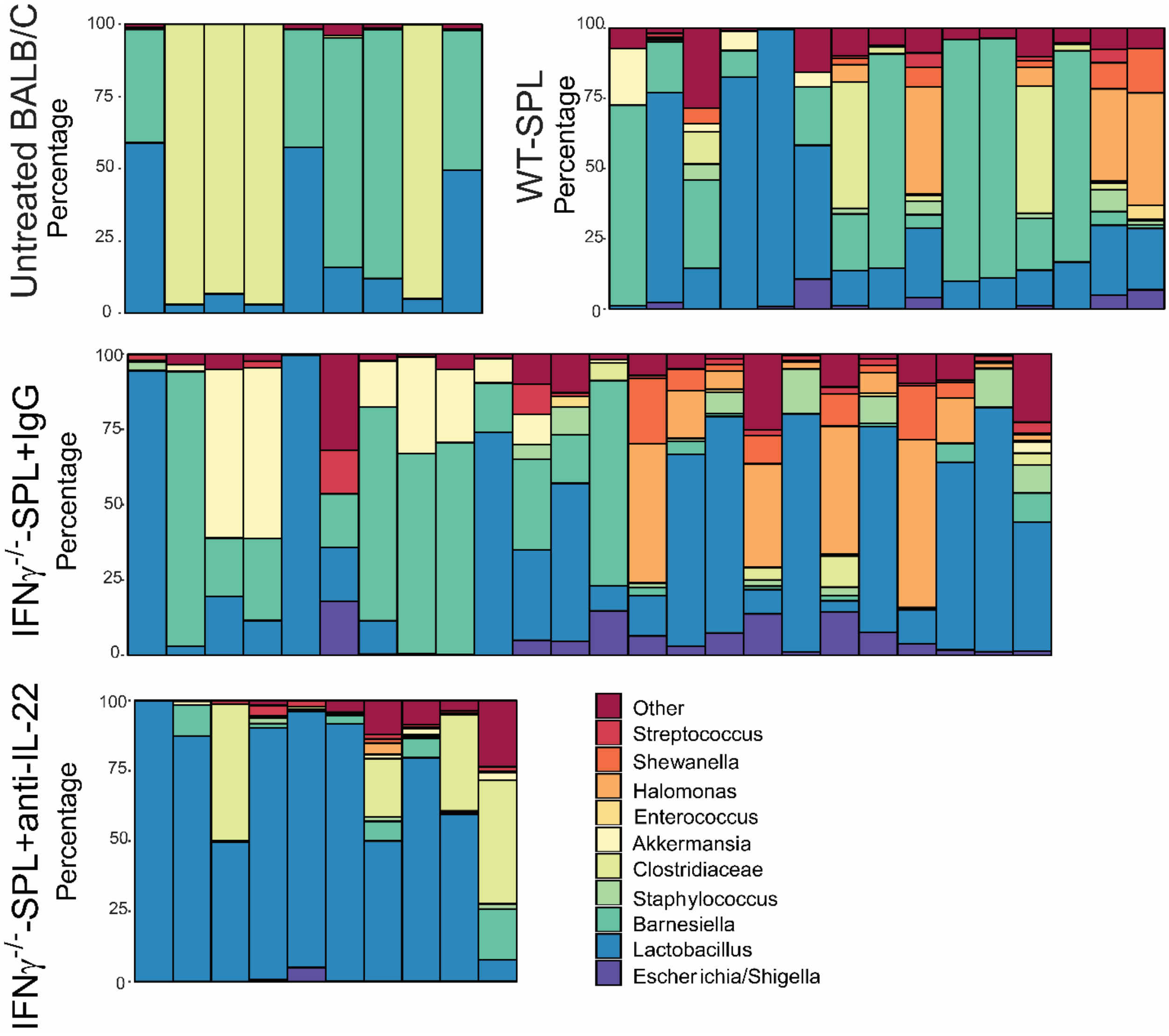

Supplementary Fig. 9. Comparison of intestinal microbiota. A supplement to Fig. 6a. Representative bacterial composition of ileal flora at the genus level.

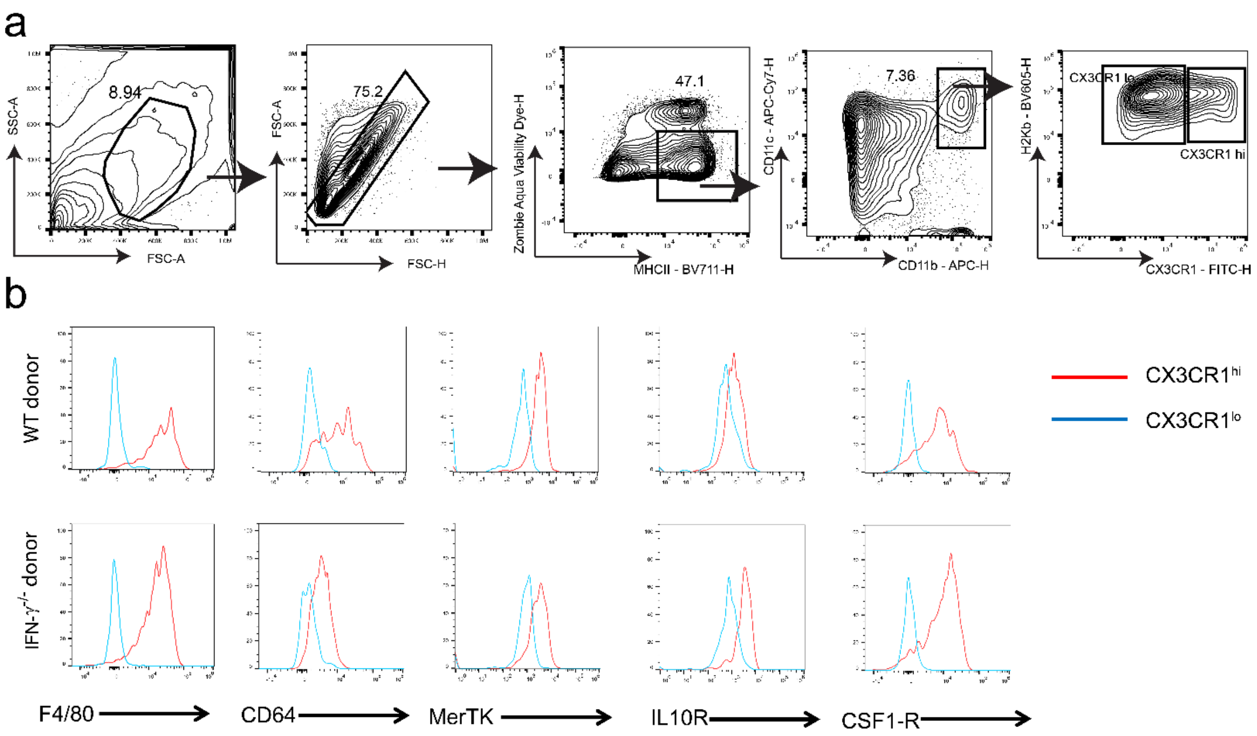

Supplementary Fig. 10. Donor-derived CX3CR1^{hi} cells have a mononuclear phagocyte phenotype. Lethally irradiated BALB/c recipients received HCT with splenocytes from WT or IFNγ$^{-/-}$ C57BL/6 donors as described in Fig. 8a. 10 days after HCT, gated CX3CR1^{hi} and CX3CR1^{lo} H-2^{b+}CD11c^{+}CD11b^{+} cells from colon tissues were analyzed for expression of F4/80, CD64, MerTK, IL-10R and CSF1-R. **(a)** Flow cytometry gating strategy. **(b)** One representative histogram is shown of 5 per group, combined from two replicate experiments.

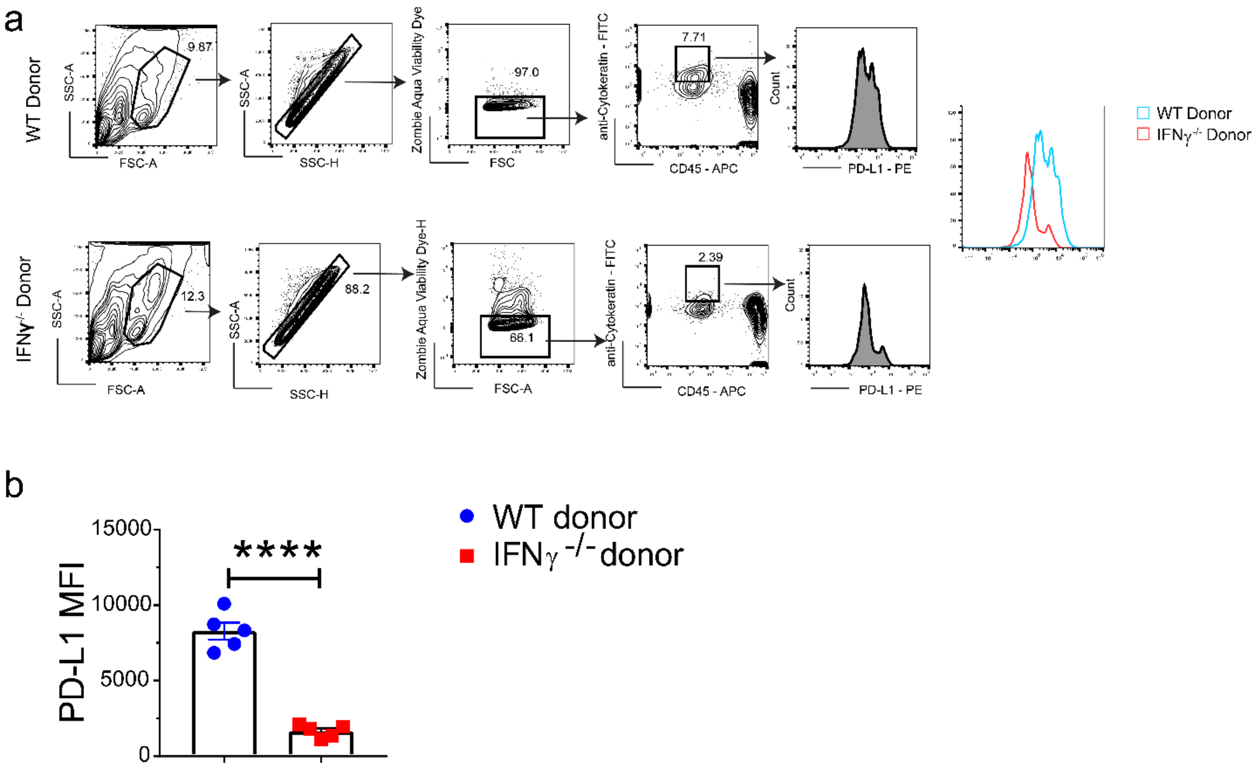

Supplementary Fig. 11. Colon epithelial cells express low levels of PD-L1 in recipients given IFN-γ$^{-/-}$-splenocytes. Lethally irradiated WT BALB/c recipients were engrafted with splenocytes (5x10^{6}) and TCD-BM (2.5x10^{6}) from IFN-γ$^{-/-}$ C57BL/6 donors and were treated with anti-CD4 mAb (500µg/mouse) on day 0 after HCT**.** On day 10 after HCT, PD-L1 expressed on colon epithelial cells (CK^{+}CD45^{-}) was measured. **(a)** Gating strategies for PD-L1 expression on colon epithelial cells. **(b)** Mean ± SE of MFI of PD-L1 is shown. n=5 per group, each dot represents one mouse, combined from two replicate experiments. Unpaired two-tailed Student's t test was used to compare means. (*****p* < 0.0001)

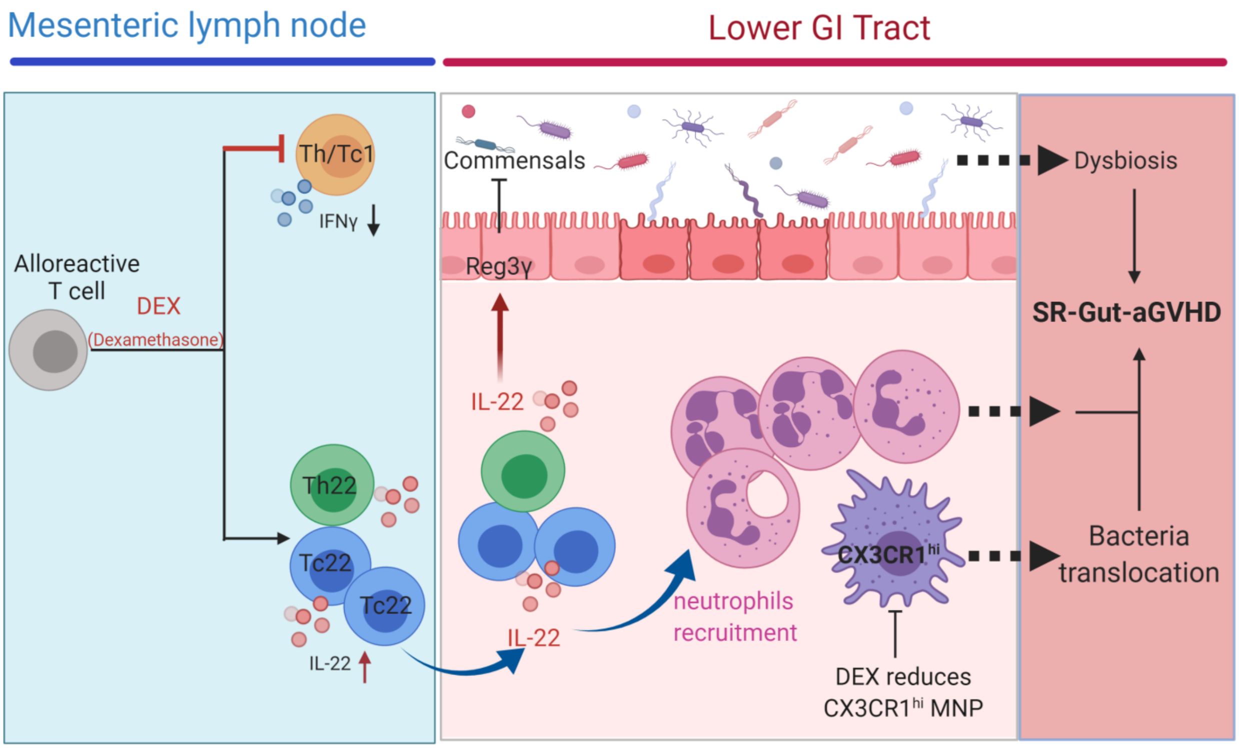

Supplementary Fig. 12: The diagram depicts the pathogenesis of (SR-Gut-aGVHD). Dexamethasone (DEX) treatment reduces IFN-γ^{+} Th/Tc1 differentiation and preferentially expands the numbers of IL-17^{-}IL-22^{+} Th/Tc22, particularly Tc22 cells. Reduction of Th/Tc1 ameliorate damage in small intestine, and expansion of Th/Tc22 cells augment damage in the lower intestinal track such as colon. The IL-22 from Th/Tc22 cells causes dysbiosis in a Reg3γ-dependent manner and augments neutrophil infiltration in the colon tissue. DEX treatment also reduces gut tissue CX3CR1^{hi} MNP that are important for controlling bacteria translocation. Dysbiosis, enhanced neutrophil infiltration, loss of CX3CR1^{hi} MNP and bacterial translocation cause full-blown SR-Gut-aGVHD in the colon.

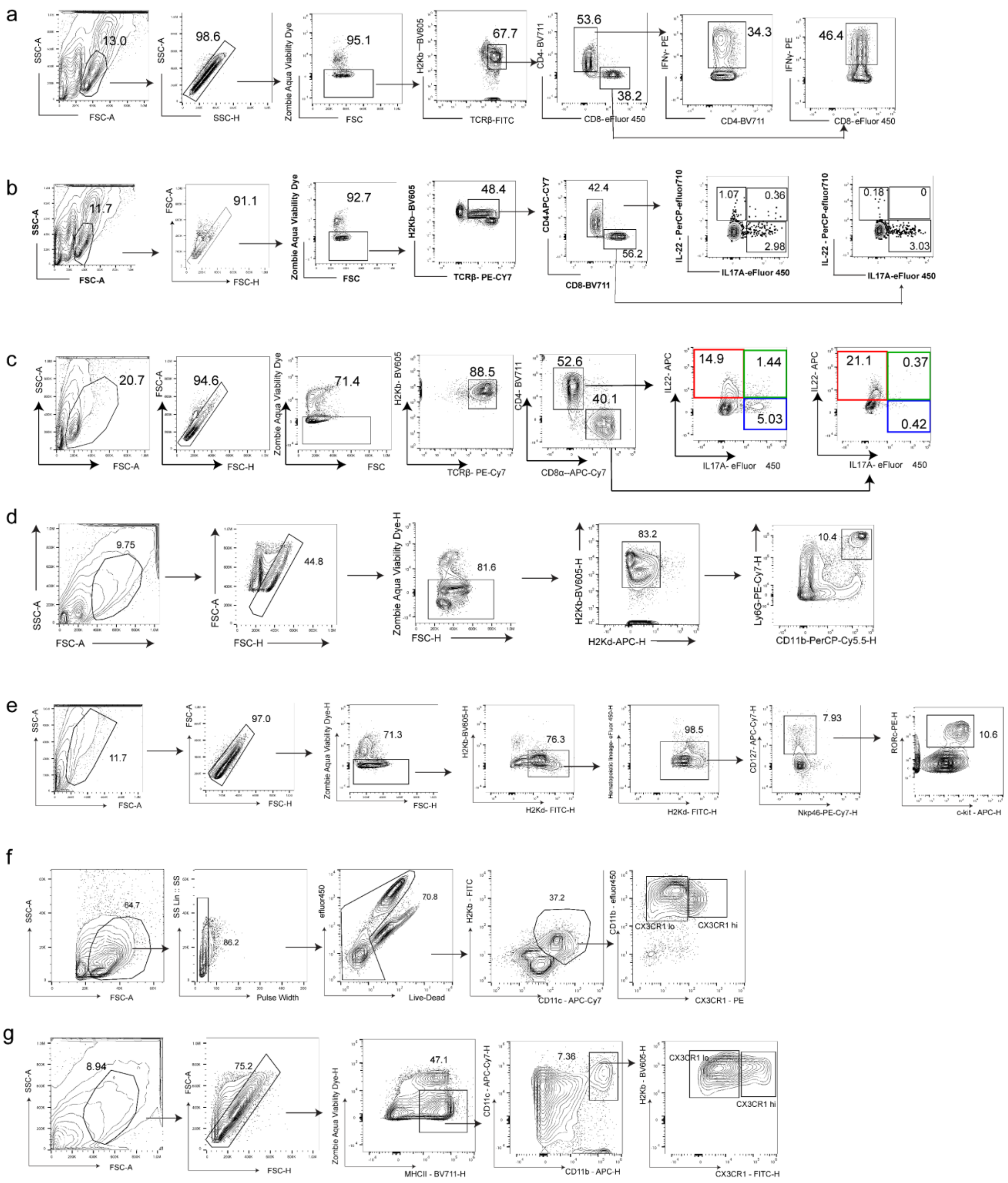

Supplementary Fig. 13: Flow cytometry gating strategies.

(a) Gating strategies for mouse Th1/Tc1 cells used in Fig. 1e and Fig. 2e.

(b) Gating strategies for mouse Th/Tc22 and Th/Tc17 cells used in Fig. 1f.

(c) Gating strategies for mouse Th/Tc22 and Th/Tc17 cells used in Fig. 2a-d, 2g, 4c, 4e-f, 5d, Supplementary Fig.7d.

(d) Gating strategies for mouse neutrophils used in Fig.5e.

(e) Gating strategies for mouse ILC3 used in supplementary Fig. 6.
(f) Gating strategies for mouse CX3CR1^{hi} MNP used in Fig.8, 9 and Supplementary Fig.10.
(g) Gating strategies for mouse CX3CR1^{hi} MNP used in Fig.10.

Supplementary table 1. Antibodies information

Antibodies	Source	Identifier	Dilutions
anti-mouse CD4 mAb	Bio-X-cell	Clone# GK1.5; Cat# BE0003-1; Lot#699918M2;	500ug/mouse
Mouse IgG	Bio-X-cell	Clone# MOPC-21; Cat# BE0083;	200ug/mouse
Anti-mouse IL-22 mAb	Genentech	Clone# 8E11; Cat# 9592; Lot# PUR163302;	200ug/mouse
Anti-mouse PD-L1	Bio-X-cell	Clone# 10F.9G2; Cat# BE0101; Lot#6154598816S1;	300ug/mouse
Anti-mouse IFN-γ mAb	Bio-X-cell	Clone# R4-6A2; Cat# BE0054; Lot#645617A1;	1mg/mouse
ChromPure Rat IgG	Jackson ImmunoResearch Laboratories	Clone#015-000-003; Cat# 012-000-003; Lot#114929;	500ug-1mg/mouse
Anti-mouse H2Kb (PE-CY7)	invitrogen	Clone# (AF6-88.5.5.3); Cat# 25-5958-82; Lot#1982659;	1:300
Anti-mouse TCRβ (PE/CY7)	Biolegend	Clone# (H57-597); Cat# 109222; Lot# B281521	1:300
Anti-mouse CD8α (efluor 450),	eBioscience	Clone# (53-6.7); Cat# 48-0081-82; Lot#1988691;	1:200
Anti-mouse CD103 (Biotin)	eBioscience	Clone# (2E7); Cat# 13-1031-85; Lot# E02662-1084;	1:300
Anti-mouse CD11b (APC)	Biolegend	Clone# (M1/70); Cat# 101211; Lot#B261577;	1:300
Anti-mouse PD-1 (APC)	eBioscience	Clone# (J43); Cat# 17-9985-82; Lot#4302240;	1:300
Streptavidin (PE-CY7),	eBioscience	Cat# 25-4317-82; Lot#E07615-1631;	1:300
Anti-mouse H2Kb (FITC)	BD Pharmingen	Clone# (AF6-88.5); Cat# 116506; Lot#7215874;	1:200
Anti-mouse IL-17A (PE)	Biolegend	Clone# (TC11-18H10.1); Cat# 506904; Lot#B247511;	1:100
Anti-mouse IL-22 (Biotin)	Biolegend	Clone# (Poly5164); Cat# 516407;	1:100

		Lot#B273228;	
Streptavidin (APC)	eBioscience	Cat# 17-4317-82;<br>Lot# E07261-1634	1:300
Anti-mouse CD11c (APC-CY7),	Biolegend	Clone#(N418);<br>Cat# 117324;<br>Lot#B237079	1:200
Anti-mouse CD103 (FITC)	invitrogen	Clone#(2E7)<br>Cat# 11-1031-82;<br>Lot#2062064	1:200
Anti-mouse CX3CR1 (PE-CY7)	Biolegend	Clone# (SA011F11);<br>Cat# 149016;<br>Lot#B216575	1:200
Anti-mouse CX3CR1 (PE)	Biolegend	Clone# (SA011F11);<br>Cat# 149006;<br>Lot#B245867	1:200
Anti-mouse CD11b (Percp-CY5.5)	Biolegend	Clone# (M1/70);<br>Cat# 101228;<br>Lot#B283049	1:300
Anti-mouse CSF1-R(BV711)	Biolegend	Clone# (AFS98)<br>Cat# 135515,<br>Lot#B251353	1:200
Anti-mouse CD64 (BV605)	Biolegend	Clone# (X54-5/7.1);<br>Cat# 139323;<br>Lot#B279592	1:200
Anti-mouse F4/80 (BV711)	Biolegend	Clone# (BM8)<br>Cat# 123147;<br>Lot#B237654	1:200
Anti-mouse MerTK (APC)	Biolegend	Clone# (2B10C42);<br>Cat# 151508;<br>Lot#B239094	1:200
Anti-mouse H2Kb (BV605)	BD Bioscience	Clone# (AF6-88.5) ;<br>Cat# 742860;<br>Lot#9217948	1:200
Anti-mouse CD8α (BUV395)	BD Bioscience	Clone# (53-6.7) ;<br>Cat# 563786;<br>Lot#8306672	1:300
Anti-mouse IL-17A (efluor450)	invitrogen	Clone# (eBio17B7) ;<br>Cat# 48-7177-82;<br>Lot#1995334	1:100
Anti-mouse IL-22 (percp-eFluor710)	Invitrogen	Clone# (1H8PWSR) ;<br>Cat#46-7221-82;<br>Lot#1924643	1:100
Anti-mouse CD103 (PE-CY7)	Biolegend	Clone#(2E7) ;<br>Cat# 121426;<br>Lot#B251087	1:200
Anti-mouse IFN gamma (PE)	eBioscience	Clone#(XMG1.2);<br>Cat# 12-7311-82;<br>Lot# E02135-1633	1:300
Anti-mouse I-A/I-E (BV711)	BD Bioscience	Clone#(M5/114.15.2);<br>Cat# 563414;	1:300

		Lot# 9210437	
Anti-mouse CD11b (APC)	ebioscience	Clone# (M1/70); Cat# 17-0112-82; Lot# E07073-1635	1:300
Anti-mouse IL-10R (PE)	Biolegend	Clone#(1B1.3a); Cat# 112706; Lot# B237811	1:200
Anti-mouse CD4 (BV711)	Biolegend	Clone# (RM4-5); Cat#100550; Lot# B273302	1:300
Anti-mouse CD8α (eFluor450)	Invitrogen	Clone# (53-6.7); Cat# 48-0081-82; Lot# 1988691	1:200
Anti-mouse CD4 (APC-CY7)	Biolegend	Clone# (GK1.5); Cat# 100414; Lot# B237980	1:300
Anti-mouse CD8α (BV711)	BD Bioscience	Clone# (53-6.7); Cat# 563046; Lot# 8325788	1:300
Monoclonal anti-Cytokeratin (FITC)	Sigma	Clone# (PCK-26); Cat# F0379; Lot# 117M4757V	1:200
Anti-mouse CD45 (APC)	eBioscience	Clone# (Ly-5); Cat# 17-0451-83; Lot# E029994	1:300
Anti-mouse CD274 (B7H1)	eBioscience	Clone# (MIH5), Cat# 12-5982-82, Lot# 4276912	1:200
Anti-mouse CD8α (APC-CY7)	Biolegend	Clone# (53-6.7); Cat# 100714; Lot# B283110	1:300
Anti-mouse TCRβ (APC-CY7)	Biolegend	Clone# (H57-597); Cat#109220; Lot# B270114	1:300
Anti-mouse hematopoietic lineage antibody cocktail (efluor450)	Invitrogen	Cat#88-7772-72, Lot#2143490	1:300
Anti-mouse NKP46(PE/CY7)	Invitrogen	Clone#(29A1.4) Cat# 25-3351-82	1:200
Anti-mouse CD127(APC/CY7)	Biolegend	Clone# (A7R34); Cat# 135040; Lot# B242148	1:200
Anti-mouse AHR(FITC)	invitrogen	Clone# (4MEJJ); Cat# 53-5925-82; Lot# 2178247	1:100
Anti-mouse CD90.2(FITC)	BD Bioscience	Clone#(30-H12); Cat# 553013; Lot# 7104844	1:200
Anti-mouse RORgT	BD Bioscience	Clone# (Q31-378);	1:100

(PE)		Cat# 562207;<br>Lot# 7201863	
Anti-human IL-22 (PE)	Invitrogen	Clone# (22URTI);<br>Cat# 12-7229-41;<br>Lot# 1938286.	1:100
Anti-human CD3(FITC)	Biolegend	Clone# (UCHT1);<br>Cat# 300440;<br>Lot# B227643	1:100
Anti-human CD4 (BV605)	Biolegend	Clone# (SK3);<br>Cat# 344645;<br>Lot# B272090	1:100
Anti-human CD8 (APC/CY7)	Biolegend	Clone# (SK1);<br>Cat# 344714;<br>Lot# B281019	1:100
Anti-human IL17A (APC)	Biolegend	Clone# (BL168);<br>Cat#512334;<br>Lot#B272155	1:100
Anti-human IFN-γ (efluor450),	Invitrogen	Clone#(4S.B3);<br>Cat# 48-3719-42;<br>Lot#4295328	1:100

Supplementary table 2. Primers information

**Names**		**Sequence (5'-3')**
Reg3γ	Forward	TTCCTGTCCTCCATGATCAAAA
	Reverse	CATCCACCTCTGTTGGGTTCA
Defensin-α3	Forward	CCCAGAAGGCTCTTCTCTTC
	Reverse	CAGCGACAGCAGAGTGTGTA
Defensin-α1	Forward	CAGGCCGTATCTGTCTCCTT
	Reverse	ATGACCCTTTCTGCAGGTTC
GAPDH	Forward	TCACCACCATGGAGAAGGC
	Reverse	GCTAAGCAGTTGGTGGTGCA
V4-V5 16S rRNA	Forward	ACACTCTTTCCCTACACGACGCTCTTCCGATCTAYTGGGYDTAAAGNG
	Reverse	GTGACTGGAGTTCAGACGTGTGCTCTTCCGATCTCCGTCAATTYHTTTREGT
