# Supplementary material for: IL-22-dependent dysbiosis and mononuclear phagocyte depletion contribute to steroid-resistant gut graft-versus-host disease in mice
Source: Nat Commun. 2021 Feb 5;12:805. doi: 10.1038/s41467-021-21133-3 (PMC7865028; doi:10.1038/s41467-021-21133-3)
Supplement: Supplementary file 3 — Reporting Summary [file 41467_2021_21133_MOESM3_ESM.pdf]

nature research

Corresponding author(s): Defu Zeng
Last updated by author(s): Qingxiao Song

Reporting Summary

Nature Research wishes to improve the reproducibility of the work that we publish. This form provides structure for consistency and transparency in reporting. For further information on Nature Research policies, see Authors & Referees and the Editorial Policy Checklist .

Statistics

For all statistical analyses, confirm that the following items are present in the figure legend, table legend, main text, or Methods section.

n/a	Confirmed	
☐	☒	The exact sample size (*n*) for each experimental group/condition, given as a discrete number and unit of measurement
☐	☒	A statement on whether measurements were taken from distinct samples or whether the same sample was measured repeatedly
☐	☒	The statistical test(s) used AND whether they are one- or two-sided<br>*Only common tests should be described solely by name; describe more complex techniques in the Methods section.*
☒	☐	A description of all covariates tested
☐	☒	A description of any assumptions or corrections, such as tests of normality and adjustment for multiple comparisons
☐	☒	A full description of the statistical parameters including central tendency (e.g. means) or other basic estimates (e.g. regression coefficient) AND variation (e.g. standard deviation) or associated estimates of uncertainty (e.g. confidence intervals)
☐	☒	For null hypothesis testing, the test statistic (e.g. *F*, *t*, *r*) with confidence intervals, effect sizes, degrees of freedom and *P* value noted<br>*Give P values as exact values whenever suitable.*
☒	☐	For Bayesian analysis, information on the choice of priors and Markov chain Monte Carlo settings
☒	☐	For hierarchical and complex designs, identification of the appropriate level for tests and full reporting of outcomes
☒	☐	Estimates of effect sizes (e.g. Cohen's *d*, Pearson's *r*), indicating how they were calculated

Our web collection on statistics for biologists contains articles on many of the points above.

Software and code

Policy information about availability of computer code

Data collection	BD FACS Diva software 8.0.1; Invitrogen Attune NxT Software V3.1; 7500 Software V2.3; Tecan i-control 1.11; PacBio Sequel ICS; Circular Consensus Sequences (CCS) module in SMRT Link (v8.0.0.80529); Illumina MiSeq platform; ViiA™ 7 Real-Time PCR System (Life Technologies) ;Agilent 2100 Bioanalyzer (Agilent Technologies);
Data analysis	Graphpad Prism8&9, Flowjo 10.0, R3.4.0, The demultiplexing and the taxonomic classification analysis of the CCS reads were carried out using SBanalyzer (v2.4-2) of Shoreline Biome (Farmington, CT) based on Athena V2 database.

For manuscripts utilizing custom algorithms or software that are central to the research but not yet described in published literature, software must be made available to editors/reviewers. We strongly encourage code deposition in a community repository (e.g. GitHub). See the Nature Research guidelines for submitting code & software for further information.

Data

Policy information about availability of data

All manuscripts must include a data availability statement. This statement should provide the following information, where applicable:

- Accession codes, unique identifiers, or web links for publicly available datasets
- A list of figures that have associated raw data
- A description of any restrictions on data availability

16S PacBio SMRT Sequencing data has been deposited in the GEO database (https://www.ncbi.nlm.nih.gov/geo/query/acc.cgi?acc=GSE159031) under the accession number GSE159031. 16S Miseq data have been deposited in GEO database (https://www.ncbi.nlm.nih.gov/geo/query/acc.cgi?acc=GSE159418) under the accession number GSE159418. The two datasets were combined in a superseries (https://www.ncbi.nlm.nih.gov/geo/query/acc.cgi?acc=GSE159419) under the accession number GSE159419. All other data supporting the findings of this study are available within the article and its supplementary information files or from the corresponding author upon reasonable request. The source data underlying Figs. 1a–f, 2a–h, 3a&d, 4a–f, 5a–k, 6a-b&d–e, 7a–c, 8a–e, 9a–h, 10a-c and Supplementary Figs. 1b, 2a-b, 3b-c, 5a-b, 6a-b, 7a-d, 8a–c, 11b are provided as a Source Data file with this publication.

Field-specific reporting

Please select the one below that is the best fit for your research. If you are not sure, read the appropriate sections before making your selection.

- [x] Life sciences
- [ ] Behavioural & social sciences
- [ ] Ecological, evolutionary & environmental sciences

For a reference copy of the document with all sections, see nature.com/documents/nr-reporting-summary-flat.pdf

Life sciences study design

All studies must disclose on these points even when the disclosure is negative.

Sample size	Sample size were determined based on previous experimental experience. For evaluation of clinical manifestation and survival, there were usually 5 mice/group in each experiment; and 2-3 replicate experiments were combined together. For in vitro mechanistic studies, there were ~ 6 individual mice/group combined from 2-3 experiments. For measuring intestinal microbiome profile, there were 2-6 mice/group combined from at least 3 replicate experiments. The precise numbers of animals and cell samples were indicated in the figure legends.
Data exclusions	We do not exclude data after protocol is established and optimized. On principle, data were only excluded for the technically failed experiments during exploring stage.
Replication	Experiments were repeated. Our data represent at least two to three independent experiments with similar results.
Randomization	For animal study, all the animals were randomly divided into different group for experiments. For human PBMC samples, samples were randomly collected.
Blinding	Investigators were blinded for disease scoring. The investigators were not blinded for cell harvest or processing due to the risk of confusion in handling. The investigator was blinded during acquisition and analysis of flow cytometry, and the same gating were applied to all samples in the given experiment, although the whole process was completed by the same person.

Reporting for specific materials, systems and methods

We require information from authors about some types of materials, experimental systems and methods used in many studies. Here, indicate whether each material, system or method listed is relevant to your study. If you are not sure if a list item applies to your research, read the appropriate section before selecting a response.

Materials & experimental systems

n/a	Involved in the study	
[ ]	[x]	Antibodies
[x]	[ ]	Eukaryotic cell lines
[x]	[ ]	Palaeontology
[ ]	[x]	Animals and other organisms
[x]	[ ]	Human research participants
[x]	[ ]	Clinical data

Methods

n/a	Involved in the study	
[x]	[ ]	ChIP-seq
[ ]	[x]	Flow cytometry
[x]	[ ]	MRI-based neuroimaging

Antibodies

Antibodies used

1. InVivoMAb anti-mouse CD4, BioXcell, Clone# GK1.5, Cat# BE0003-1; Lot#699918M2; 500ug/mouse.
2. InVivoMAb anti-mouse PD-L1 (B7-H1), BioXcell, Clone# 10F.9G2,Cat# BE0101,Lot#6154598816S1; 300ug/mouse.
3. InVivoMAb anti-mouse IFNg, BioXcell, Clone# ,Cat# BE0054, Lot#645617A1; 1mg/mouse.
4. InVivoMAb mouse IgG1 isotype control, BioXcell, clone# MOPC-21,Cat# BE0083; 200ug/mouse.
5. Anti-mouse IL-22 mAb, Genentech (South San Francisco, California). clone#8E11, Cat# 9592,Lot# PUR163302; 200ug/mouse.
6. ChromPure Rat IgG, Jackson ImmunoResearch Laboratories, Inc (West Grove, PA, USA). Cat#(012-000-003) Clone#015-000-003,Lot#114929; 500ug-1mg/mouse.
7. Anti-mouse H2Kb (PE-CY7), invitrogen, Clone# (AF6-88.5.5.3), Cat# 25-5958-82; Lot#1982659; 1:300.
8. Anti-mouse TCRβ (PE-CY7), Biolegend, Clone# (H57-597), Cat# 25-5961-82;Lot# B281521; 1:300.
9. Anti-mouse CD8α (efluor 450), eBioscience, Clone# (53-6.7), Cat# 48-0081-82,Lot#1988691; 1:200.
10. Anti-mouse CD103 (Biotin), eBioscience, Clone# (2E7), Cat# 13-1031-85, Lot#E02662-1084; 1:300.
11. Anti-mouse CD11b (APC), Biolegend, Clone# (M1/70), Cat# 101211; Lot#B261577; 1:300.
12. Anti-mouse PD-1 (APC), eBioscience, Clone# (J43), Cat# 17-9985-82;Lot#4302240; 1:300.
13. Streptavidin (PE-CY7), eBioscience, Cat# 25-4317-82, Lot#E07615-1631; 1:300.
14. Anti-mouse H2Kb (FITC), BD Pharmingen, Clone# (AF6-88.5), Cat# 116506, Lot#7215874; 1:200.
15. Anti-mouse IL-17A (PE), Biolegend, Clone# (TC11-18H10.1) , Cat# 506904, Lot#B247511; 1:100.
16. Anti-mouse IL-22 (Biotin), Biolegend, Clone# (Poly5164) , Cat# 516407;Lot#B273228; 1:100.

17. Streptavidin (APC), eBioscience, Cat# 17-4317-82; Lot# E07261-1634; 1:300.
18. Anti-mouse CD11c (APC-CY7) , Biolegend, Clone#(N418) , Cat# 117324, Lot#B237079; 1:200.
19. Anti-mouse CD103 (FITC), invitrogen, Clone#(2E7) , Cat# 11-1031-82;Lot#2062064; 1:200.
20. Anti-mouse CX3CR1 (PE-CY7), Biolegend, Clone# (SA011F11), Cat# 149016, Lot#B216575; 1:200.
21. Anti-mouse CX3CR1 (PE), Biolegend, Clone# (SA011F11), Cat# 149006, Lot#B245867; 1:200.
22. Anti-mouse CD11b (Percp-CY5.5), Biolegend, Clone# (M1/70), Cat# 101228;Lot#B283049; 1:300.
23. Anti-mouse CSF1-R(BV711), Biolegend, Clone# (AFS98) , Cat# 135515, Lot#B251353; 1:200.
24. Anti-mouse CD64 (BV605), Biolegend, Clone# (X54-5/7.1) , Cat# 139323,Lot#B279592; 1:200.
25. Anti-mouse F4/80 (BV711), Biolegend, Clone# (BM8) , Cat# 123147,Lot#B237654; 1:200.
26. Anti-mouse MerTK (APC), Biolegend, Clone# (2B10C42) , Cat# 151508, Lot#B239094; 1:200.
27. Anti-mouse H2Kb (BV605), BD Bioscience, Clone# (AF6-88.5) , Cat# 742860, Lot#9217948; 1:200.
28. Anti-mouse CD8α (BUV395), BD Bioscience, Clone# (53-6.7) , Cat# 563786; Lot#8306672; 1:300.
29. Anti-mouse IL-17A (efluor450), invitrogen, Clone# (eBio17B7) ,Cat# 48-7177-82; Lot#1995334; 1:100.
30. Anti-mouse IL-22 (percp-eFluor710), Invitrogen, Clone# (1H8PWSR) ,Cat#46-7221-82, Lot#1924643; 1:100.
31. Anti-mouse CD103 (PE-CY7), Biolegend, Clone#(2E7) , Cat# 121426;Lot#B251087;1:200.
32. Anti-mouse IFN gamma (PE), ebioscience, Clone#(XMG1.2), Cat# 12-7311-82, Lot# E02135-1633; 1:300.
33. Anti-mouse I-A/I-E (BV711), BD Bioscience, Clone#(M5/114.15.2), Cat# 563414, Lot# 9210437; 1:300.
34. Anti-mouse CD11b (APC), ebioscience, Clone# (M1/70), Cat# 17-0112-82, Lot# E07073-1635; 1:300.
35. Anti-mouse IL-10R (PE), Biolegend, Clone#(1B1.3a), Cat# 112706, Lot# B237811; 1:200.
36. Anti-mouse CD4 (BV711), Biolegend, Clone# (RM4-5), Cat#100550, Lot# B273302; 1:300.
37. Anti-mouse CD8 (eFluor450), Invitrogen, Clone# (53-6.7), Cat# 48-0081-82, Lot# 1988691; 1:200.
38. Anti-mouse CD4 (APC-CY7), Biolegend, Clone# (GK1.5), Cta# 100414, Lot# B237980; 1:300.
39. Anti-mouse CD8 (BV711), BD Bioscience, Clone# (53-6.7), Cat# 563046, Lot# 8325788; 1:300.
40. Monoclonal anti-Cytokeratin (FITC), Sigma, Clone# (PCK-26), Cat# F0379, Lot# 117M4757V; 1:200.
41. Anti-mouse CD45 (APC), eBioscience, Clone# (Ly-5), Cat# 17-0451-83, Lot# E029994; 1:300.
42. Anti-mouse CD274 (B7H1), eBioscience, Clone# (MIH5), Cat# 12-5982-82, Lot# 4276912; 1:200.
43. Anti-mouse CD8 (APC-CY7), Biolegend, Clone# (53-6.7), Cat# 100714, Lot# B283110; 1:300.
44. Anti-mouse TCRbeta (APC-CY7), Biolegend, Clone# (H57-597), Cat#109220, Lot# B270114; 1:300.
45. Anti-mouse hematopoietic lineage antibody cocktail efluor450, Cat#88-7772-72, Lot#2143490; 1:300.
46. Anti-mouse NKP46(PE/CY7), Invitrogen, Clone#(29A1.4),Cat# 25-3351-82; 1:200.
47. Anti-mouse AHR(FITC), invitrogen, Clone#(4MEJJ) , Cat#53-5925-82, Lot# 2178247.1:200.
48. Anti-mouse CD127(APC/CY7), Biolegend, Clone#(A7R34), Cat#135040, Lot#B242148.1:200.
49. Anti-mouse CD90.2(FITC), BD Bioscience, Clone#(30-H12);Cat# 553013;Lot# 7104844, 1:200.
50. Anti-mouse RORgT (PE), BD Bioscience, Clone# (Q31-378), Cat#562207, Lot#7201863. 1:100.
51. Anti-human IL-22 (PE), Invitrogen, Clone# (22URTI) , Cat# 12-7229-41, Lot# 1938286.1:100.
52. Anti-human CD3(FITC), Biolegend, Clone#(UCHT1), Cat#300440, Lot#B227643. 1:100.
53. Anti-human CD4 (BV605), Biolegend, Clone# (SK3), Cat#344645, Lot#B272090. 1:100.
54. Anti-human CD8 (APC/CY7), Biolegend, Clone# (SK1), Cat#344714, Lot#B281019. 1:100.
55. Anti-human IL17A (APC), Biolegend, Clone#(BL168) , Cat#512334, Lot#B272155. 1:100.
56. Anti-human IFNgamma (efluor450), Invitrogen, Clone#(4S.B3) , Cat# 48-3719-42; Lot#4295328. 1:100.

Validation

All the antibody were validated by manufacturer, and the information about the validation can be found on the manufacturer's website through the links below: https://www.biolegend.com/; https://www.thermofisher.com/us/en/home.html; https://www.bdbiosciences.com/en-us; https://www.sigmaaldrich.com/united-states.html; https://bxcell.com/;

Animals and other organisms

Policy information about studies involving animals; ARRIVE guidelines recommended for reporting animal research

Laboratory animals

BALB/c (H-2d) (Stock# 555) and C57BL/6 (H-2b) (Stock# 556) mice were purchased from the National Cancer Institute (Frederick, MD). PD-L1-/- BALB/c (H-2d) breeders were provided by Dr. Lieping Chen (Yale University)59, 60. Reg3γ-/- C57BL/6 (H-2b) breeders were provided by Dr. James Ferrara (Mount Sinai Hospital, NY)33. IFN-γ-/- C57BL/6 (H-2b) (Stock# 002287), IFN-γ-/- BALB/c (H-2d) (Stock# 002286), CB6F1 (H-2b/d) (Stock# 100007), IL-22-/- C57BL/6 (H-2b) (Stock# 027524) , B6(Cg)-Rorctm3Litt/J (Stock# 008771), B6.Cg-Tg(Cd4-cre)1Cwi/BfluJ (Stock# 022071), Rag2-γc- (Stock# 014593) and B6.129P2(Cg)-Cx3cr1tm1Litt/J (Stock# 005582) were purchased from the Jackson Laboratory (Bar Harbor, ME). B6.129P2(Cg)-Cx3cr1tm1Litt/J mice were mated with WT C57BL/6 mice to generate CX3CR1+/- mice. B6(Cg)-Rorctm3Litt/J mice were mated with B6.Cg-Tg(Cd4-cre)1Cwi/BfluJ mice to generate T- RORγt-/- C57BL/6 (H-2b) mice. RORγt-/- C57BL/6 (H-2b) mice were provided by Dr. Zuoming Sun (City of hope, Duarte)61. IFN-γ-/- C57BL/6 mice were crossing with RORγt-/- C57BL/6 mice to generate the IFN-γ-/-/RORγt-/- C57BL/6 (H-2b) mice. IFN-γ−/−IL-17−/− C57BL/6 (H-2b) mice were generated by crossing IFN-γ−/− mice with IL-17−/− mice52. H-2Kb+IA-IE- BALB/c mice were generated by backcrossing MHCII-/- C57BL/6 62 mice into WT BALB/c mice for more than 12 generations The experimental mice were male and were used at 8-12 weeks. Animal breeding and experiments were performed in separate specific pathogen-free rooms, and control and experimental mice were kept in separate cages in the same room at City of Hope Animal Research Center (ARC). 12 light/12 dark cycle, temperatures of 68-75°F with 30-70% humidity are used. All procedures were performed in the animal facility in compliance with a protocol approved by the City of Hope Institutional Animal Care and Use Committee (IACUC) under IACUC protocol 03008. All Mice were euthanized by CO2 from compressed gas cylinders, and we complied with all the ethical regulations.

Wild animals	This study did not involve wild animals.
Field-collected samples	This study did not involve the use of the field-collected samples.
Ethics oversight	All mice were maintained in a pathogen-free room in the City of Hope Animal Research Center. All animal protocols were approved by the City of Hope Institutional Animal Care and Use Committee (IACUC).

Note that full information on the approval of the study protocol must also be provided in the manuscript.

Flow Cytometry

Plots

Confirm that:

- [x] The axis labels state the marker and fluorochrome used (e.g. CD4-FITC).
- [x] The axis scales are clearly visible. Include numbers along axes only for bottom left plot of group (a 'group' is an analysis of identical markers).
- [x] All plots are contour plots with outliers or pseudocolor plots.
- [x] A numerical value for number of cells or percentage (with statistics) is provided.

Methodology

Sample preparation	-> For cell sorting:<br>Single-cell suspensions were prepared from spleen by crushing on 70m cell strainers. Cells were run<br>through cell strainer and red blood cells were lysed in Red blood lysis buffer for 5 min. Total CD11c+ cells were isolated by using antimouse CD11c beads(Miltenyi Biotec). Specific MHC2+CD11b+CX3CR1hi cell populations were then sorted on FACSAria II SORP.<br>-> For cell analysis:<br>Cell surface and intracellular staining were performed on single-cell suspensions. For intracellular staining, cell were fixed and permeabilized using the Foxp3/Transcriptional Factor Staining Buffer Set (Thermo Fisher).
Instrument	CyAn Immunocytometry system (DAKO Cytomation, Fort Collins, CO), Attune NxT Flow Cytometer (ThermoFisher Scientific) and BD LSRFortessa (Franklin Lakes, NJ)
Software	FACSDiva for collection and FlowJo (version 10) for analysis
Cell population abundance	1-3 million mononuclear cells were used for flow cytometry staining. Purity was assessed by staining the general markers for T cell and other mononuclear cells.
Gating strategy	Relevant gating strategies shown in Supplementary figure 13.

- [x] Tick this box to confirm that a figure exemplifying the gating strategy is provided in the Supplementary Information.
